# A Cross-Sectional Study on Client Satisfaction of Opioid Substitution Therapy (OST) in a Tertiary Care Hospital of Punjab, India

**DOI:** 10.7759/cureus.99665

**Published:** 2025-12-19

**Authors:** Jaskaran Singh, Rajnish Raj, Bhavneesh Saini, Priyanka Bansal, Harshdeep Kaur, Tejasvi Kainth, Sikandar Saeed, Sakshi Prasad, Sasidhar Gunturu

**Affiliations:** 1 Psychiatry and Behavioral Sciences, Nassau University Medical Center, East Meadow, USA; 2 Psychiatry, Government Medical College and Rajindra Hospital, Patiala, IND; 3 Psychiatry, Guru Gobind Singh Medical College, Faridkot, IND; 4 Medicine, Government Medical College and Rajindra Hospital, Patiala, IND; 5 Psychiatry, BronxCare Health System, Bronx, USA

**Keywords:** addiction, bpn, buprenorphine, client satisfaction, opioid substitution therapy, opioid use, ost, punjab, quality, substance use

## Abstract

Introduction: Opioid substitution therapy (OST) program under the National Aids Control Organization (NACO), India, is a harm-reduction approach targeting injectable opioid use. However, this ‘one-size-fits-all’ approach may potentially deter client satisfaction. Scant data on service quality in India warranted the current research.

Methods: This was a cross-sectional study in one OST center at a tertiary-care hospital in Punjab, India. Socio-demographic details of registered OST clients were collected. Client satisfaction was assessed using the GGZ (Geestelijke Gezondheidszorg; Dutch: mental healthcare) Thermometer.

Results: A total of 142 clients participated. Mean quality score was 7.6±1.5 (satisfactory). Positive rating was given by 93.7%, 46.5%, 61.3%, and 97.2% of clients for information, decision making, care provider, and treatment result domains, respectively. An open question regarding quality revealed ‘requirement of advanced doses’ by 24.6% and ‘difficulty in daily visits’ by 11.3%. GGZ score negatively correlated with current buprenorphine+naloxone (BPN) dose (r=-0.17, p=0.046). Those with a <6 score had a longer duration on OST (p=0.011), which positively correlated with starting BPN dose (r=0.43, p<0.001).

Conclusion: It was concluded that the center performed satisfactorily at providing information and treatment results, but lacked in client participation and caregiver standards. Individual tailoring of treatment and enhancing on-site psychosocial support is needed to improve quality.

## Introduction

The National Survey on Extent, Pattern and Trends of Drug Abuse in India, 2004, quotes the number of opioid users to be two million, out of which half a million are dependent [1]. The Punjab State Opioid Survey, 2015, elicited 232,856 persons with opioid dependence [2].

Of the numerous treatment options available, the opioid substitution therapy (OST) program is one such government-promoted harm-reduction approach [3]. As per the National Aids Control Organization (NACO) report 2018-19, more than 28 centres in Punjab provide coverage to over 8000 substance users [4]. Unlike Western countries, where there is a stepped-care model, India holds a “one-size-fits-all” approach, giving lesser preference to individual client preferences [1]. Moreover, the administration's focus on monitoring and evaluation has been on audits that are a one-way measure [5], with minimal user involvement.

Client satisfaction is indispensable, so the present scenario calls for an evaluation of OST service quality, ultimately aimed at improving service delivery, resource allocation, and health policy. Considering the scarcity of monitoring and evaluation data from India, the current study aimed to: (i) assess client satisfaction with OST services, (ii) evaluate service quality across the mental health domains, (iii) examine sociodemographic and clinical factors associated with satisfaction, and (iv) explore, as a secondary objective, the role of personality traits in clients’ perceptions of treatment.

## Materials and methods

This was a cross-sectional study in the OST centre, Government Medical College and Rajindra Hospital, Patiala, Punjab, India. The study was designed as a pragmatic service-evaluation exercise. It was approved by the Institutional Ethical Committee, Government Medical College, Patiala, Punjab, India (approval number: Trg9(310)2022/38566).

Study population

Eligibility Criteria

The study included clients aged 18-60 who met International Classification of Diseases, Tenth Revision (ICD-10) criteria for opioid dependence (F11.2), were enrolled for at least six months, and provided written informed consent. Clients who failed to consent, had severe organic ailments, or were blacklisted at high risk for diversion in staff records were excluded.

Sample Size and Sampling

All consecutive eligible clients attending the OST center during the study month were approached for participation. Recruitment was conducted by trained psychiatry residents using a standardized invitation script. The number of clients who declined or were unavailable was recorded where possible, though detailed refusal data could not be systematically collected. 

Based on data for the previous month of the study, September 2021, 167 clients presented for follow-up, all of whom were initially enrolled via purposive sampling. Following scrutiny of the inclusion/exclusion criteria, the final size was 142. 

Operational guidelines

The OST standard operating procedures [5] and clinical practice guidelines [6] issued by NACO, India, were followed. These include a preparatory phase where clients referred by outreach services like targeted intervention (TI) workers or healthcare providers are informed and assessed for suitability for OST. OST has an induction, maintenance, and termination phase where clients are started on sublingual buprenorphine+naloxone (BPN) in either 2 mg/0.5 mg or 0.4 mg/0.1 mg fixed-dose formulation, depending upon clinical signs/symptoms of dependence and withdrawal. Once a stabilization stage is achieved (usually within three to seven days), BPN is maintained for months to years. Take-home or advanced doses are given in exceptional circumstances, such as civil unrest, emergencies after COVID-19, or staff unavailability. Psychosocial intervention, such as brief intervention, motivation enhancement, and relapse prevention, is provided to all. Lastly, termination is planned after treatment goals are met, which includes good psychosocial and occupational functioning.

Study procedure

Written informed consent was taken. Questionnaires were interviewer-administered by psychiatry residents trained for two days in standardized administration procedures, use of scripted prompts, and data-quality checks. Uniformity across interviewers was maintained through daily supervision by the senior investigator. A semi-structured OST proforma was filled out for socio-demographic and drug-related details. Client satisfaction and OST Quality were assessed on the GGZ (Geestelijke Gezondheidszorg; Dutch: mental healthcare) Thermometer for appreciation by clients [7]. After preliminary GGZ findings suggested heterogeneity in satisfaction patterns, an exploratory post-hoc Big Five Inventory-brief version (BFI-10) [8] assessment was conducted among clients who were present during the subsequent data-collection window and provided additional consent. This subset was not randomly selected and, therefore, may have introduced selection bias. Missing data were minimal and handled using complete-case analysis. Nonparametric tests were selected owing to non-normal score distributions and ordinal scale properties, although formal assumption testing was not conducted. Data collection was done in October 2021 during working hours every day of the month until complete enrolment.

Instruments

Socio-Demographic Proforma

A semi‑structured proforma containing socio-demographic and drug-related details was used. This included categories of age, age of onset, marital status and partner details, residence, distance from OST centre, education, occupation, referral status, blood-borne viral marker status, psychiatric history, and substance(s) consumed other than opioids. OST-related details included the initial starting and current BPN dose, duration on BPN, number of advance doses, and any client comments/ suggestions related to OST.

GGZ Thermometer for Appreciation by Clients

This is a self-rated questionnaire, freely available for research use, containing 16 yes-no questions, assessing four client satisfaction domains: (i) Appreciation of Information, (ii) Appreciation of Decision Making, (iii) Appreciation of Healthcare Provider, and (iv) Treatment Results (three, three, four, and six questions, respectively).

A positive rating (‘yes’ response) by ≥80% clients to each question is optimal. The scale further consists of an overall satisfaction score with a cut-off score of 6/10 for satisfactory quality, an open question for suggestions on quality, and a miscellaneous question. The scale was translated into the Punjabi language for use by the study population. This was done by three experienced psychiatrists well-versed in English and Punjabi. The investigators then reviewed the versions, and the discrepancies were resolved by consensus. A literary expert then cross-checked both versions. However, the translation process did not include formal back-translation or full cross-cultural validation. The emphasis was on content and meaning rather than on maintaining structure or language. Therefore, the Punjabi version should be considered a preliminary adaptation.

A pilot study was done in 30 clients (distinct from the study sample) to assess its reliability and validity in the study population. Face validity was established by the department's consultants. Reliability was assessed using Cronbach’s Alpha, which was 0.81. For this study, a positive ‘question rating’ to all questions in that domain was considered a positive ‘domain rating’. Further, separate from the overall satisfaction score, i.e., ‘GGZ score’, an overall positive ‘GGZ rating’ was considered when a positive rating to ≥80% questions (13 out of 16 questions) was given.

BFI-10

This is a brief version of the Big Five Personality Inventory, having 10 questions, two questions for each of the five personality traits: neuroticism, extraversion, openness, conscientiousness, and agreeableness. The BFI-10 is an open-access instrument that can be used for non-commercial research.

Comparison groups (observations made after the pilot study)

**Figure 1:**
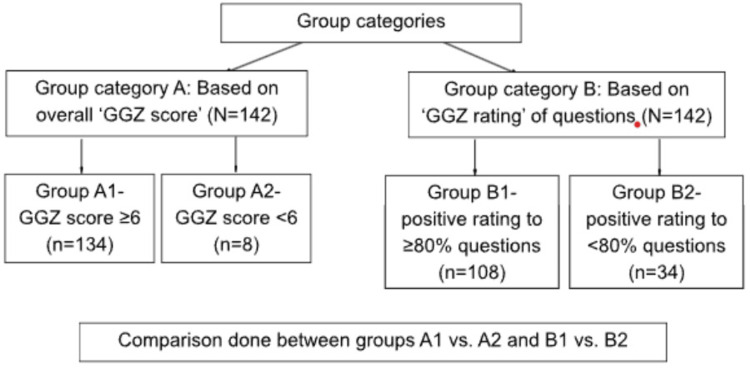
Comparison group categories GGZ (Geestelijke Gezondheidszorg): Mental Healthcare Thermometer for Appreciation by Clients [7]

Clients giving an overall high rating (e.g., 9 or 10) on the GGZ scale were found to have answered negatively, i.e., ‘No’ on more than a few questions on the scale (Q1-Q16). Clients giving an overall lower rating (e.g., six or seven) onthe GGZ scale were found to have answered positively, i.e., ‘Yes’ on most individual scale questions (Q1-Q16). It was thus hypothesized that indicators of good quality varied among individuals. A lack in one or more domains did not equate to dissatisfaction for all clients. Hence, for the current study, two group-categories for analysis were made, as detailed in Figure 1.

Statistical analysis

The IBM SPSS Statistics for Windows, version 23.0 (Released 2015; IBM Corp., Armonk, New York, United States) software was used. Categorical variables were compared using Pearson’s Chi-square test, with Bonferroni correction for multiple comparisons. Fisher’s exact test was used where the expected cell count (≥20% cells) was <5. Continuous variables were compared using the Mann-Whitney U test for two groups and the Wilcoxon one-sample Signed-rank test for one group. Correlation analysis was done using Kendall’s tau b test. ‘P’ values <0.05 were considered significant.

## Results

Table 1 shows the socio-demographic variables of OST clients. They had a mean age of 40.4 ± 10.0 years; all were male, most were married (70.4%), with regular partners (71.1%), educated till matriculation (28.9%), employed full time (59.0%), and referred for treatment by TI workers (76.8%). Of the clients, 6.3% were found positive for any viral marker(s), 7.7% had a positive psychiatric history, and 9.2% had a co-morbid substance use. Based on groups made as per GGZ score (Group category A) and GGZ rating (Group category B), no socio-demographic differences were found except that persons giving positive ratings to ≥80% questions were more self-employed (40.7%), while those giving <80% were more unemployed (20.6%). 

**Table 1:** Comparison of socio-demographic characteristics (N=142) * Chi-square test used; Fisher’s exact test used where ≥20% expected cell counts were less than 5; Mann-Whitney U test used for continuous variables. ^†^Others- separated/ divorced/ widowed; TI- Targeted Intervention; HHH- HIV/ HBsAg/ HCV. ^‡^Each subscript letter (a and b) denotes a subset of Occupation categories whose proportions differ significantly from the other at .05 level on Bonferroni correction.

Parameter	Category	Total sample (N=142)	Group category A*		Group category B*	
			GGZ score <6, (n=8)	GGZ score ≥6, (n=134)	‘Yes’ for ≥80% questions, (n=108)	‘Yes’ for <80% questions, (n=34)
Age	Mean ± SD (median)	40.4 ± 10.0 (39.0)	42.4 ± 9.8 (40.0)	40.3 ± 10.1 (39.0)	40.9 ± 10.3 (38.0)	39.0 ± 8.9 (39.5)
p value		-	0.538		0.381	
Age of onset of substance	Mean ± SD (Median)	23.4 ± 6.0 (21.0)	24.0 ± 5.8 (24.5)	23.4 ± 6.0 (21.0)	23.4 ± 6.0 (21.0)	23.4 ± 5.8 (21.5)
p value		-	0.634		0.899	
Marital Status	Unmarried, n (%)	37 (26.1)	2 (25)	35 (26.1)	28 (25.9)	9 (26.1)
	Married, n (%)	100 (70.4)	6 (75)	94 (70.1)	75 (69.4)	25 (73.5)
	Others†, n (%)	5 (3.5)	0	5 (3.7)	5 (4.6)	0
p value		-	1.000		0.610	
Regular partner	Yes, n (%)	101 (71.1)	6 (75)	95 (70.9)	76 (70.4)	25 (73.5)
	No, n (%)	41 (28.9)	2 (25)	39 (29.1)	32 (29.6)	9 (26.5)
p value		-	1.000		0.723	
Occupation	Unemployed, n (%)	15 (10.6)	0	16 (11.9)	9 (8.3)_a_	7 (20.6)_b_
	Self-employed, n (%)	42 (29.6)	4 (50)	47 (35.1)	44 (40.7)_a_	7 (20.6)_b_
	Part-time employed, n (%)	1 (0.7)	3 (37.5)	30 (22.4)	26 (24.1)	7 (20.6)
	Full-time employed, n (%)	84 (59.2)	1 (12.5)	41 (30.6)	29 (26.9)	13 (38.2)
p value		-	0.469		0.054‡	
Education	Illiterate, n (%)	34 (23.9)	1 (12.5)	33 (24.6)	22 (20.4)	12 (35.3)
	Primary, n (%)	26 (18.3)	2 (25)	24 (17.9)	24 (22.2)	2 (5.9)
	Middle, n (%)	31 (21.8)	3 (37.5)	28 (20.9)	25 (23.1)	6 (17.6)
	Matriculation, n (%)	41 (28.9)	1 (12.5)	40 (29.9)	30 (27.8)	11 (32.4)
	≥ Graduation, n (%)	10 (7)	1 (12.5)	9 (6.7)	7 (6.5)	3 (8.8)
p value		-	0.491		0.140	
Referred by	TI worker†, n (%)	109 (76.8)	8 (100)	101 (75.4)	82 (75.9)	27 (79.4)
	Self, n (%)	26 (18.3)	0	26 (19.4)	21 (19.4)	5 (14.7)
	Others, n (%)	7 (4.9)	0	7 (5.2)	5 (4.6)	2 (5.9)
p value		-	0.573		0.802	
Viral Markers	Positive (HHH)†, n (%)	9 (6.3)	1 (12.5)	8 (6)	8 (7.4)	1 (2.9)
	Negative, n (%)	133 (93.7)	7 (87.5)	126 (94)	100 (92.6)	33 (97.1)
p value		-	0.416		0.687	
Psychiatric History	Present, n (%)	11 (7.7)	0 (0)	11 (8.2)	10 (9.3)	1 (2.9)
	Absent, n (%)	131 (92.3)	8(100)	123 (91.8)	98 (90.7)	33 (97.1)
p value		-	1.000		0.460	
Comments/ suggestions	Add-on Medication, n (%)	2 (1.4)	0	2 (1.5)	2 (1.8)	0
	Advanced doses, n (%)	35 (24.6)	3 (37.5)	32 (23.9)	26 (24.1)	9 (26.5)
	Daily visit difficult, n (%)	16 (11.3)	1 (12.5)	15 (11.2)	11 (10.2)	5 (14.7)
	Long waiting, n (%)	4 (2.8)	1 (12.5)	3 (2.2)	4 (3.7)	0
	Regular counseling needed, n (%)	3 (2.1)	0	3 (2.2)	1 (0.9)	2 (5.9)
	Uncrushed tablets/ Divided doses, n (%)	12 (8.4)	1 (12.5)	11 (8.2)	9 (8.3)	3 (8.8)
	Want to stop (long duration of Rx) , n (%)	2 (1.4)	0	2 (1.5)	2 (1.8)	0
	Total, n (%)	74 (52.1)	6 (75)	68 (50.8)	55 (50.9)	19 (55.9)
p value		-	0.796		0.589	
Add-on substance use	Alcohol, n (%)	7 (4.9)	0	7 (5.2)	5 (4.6)	2 (5.9)
	Opioids, n (%)	2 (1.4)	0	2 (1.5)	2 (1.8)	0
	Cannabis, n (%)	2 (1.4)	1 (12.5)	1 (0.8)	1 (0.9)	1 (2.9)
	Nicotine, n (%)	2 (1.4)	1 (12.5)	1 (0.8)	0	2 (5.9)
	Total, n (%)	13 (9.2)	2 (25)	11 (8.2)	8 (7.4)	5 (14.7)
p-value			0.192		0.380	

Table 2 shows an overall good rating of the OST center (mean GGZ score 7.6 ± 1.5). Unsatisfied clients (GGZ score <6) had a significantly (p=0.011) longer duration of being on BPN (6.8 ± 1.8 years) compared to satisfied clients (GGZ score ≥6). No intra-group differences were found based on Initial and current BPN dose or the distance from the OST center. Ten advance doses were given in the month, which shows that most clients followed up every third day at the center. Regarding comments/suggestions for the OST center (Table 1), 24.6% (n=35) of clients felt the need for advance doses, and 11.3% (n=16) felt that daily visits to the OST center were difficult.

**Table 2:** Comparison of mean score variables *Mann-Whitney U test used. BPN: buprenorphine+naloxone; OST: opioid substitution therapy; GGZ: Geestelijke Gezondheidszorg (Dutch, Mental Healthcare) Thermometer for Appreciation by Clients [6]

Parameter	Overall sample; N=142	Group category A		p value*	Group category B		p value*
		GGZ score <6 (n=8)	GGZ score ≥6 (n=134)		‘Yes’ for ≥80% questions (n=108)	‘Yes’ for <80% questions (n=34)	
	Mean ± SD (Median)	Mean ± SD (Median)	Mean ± SD (Median)		Mean ± SD (Median)	Mean ± SD (Median)	
Initial BPN dose (mg)	5.6 ± 2.4 (6.0)	6.5 ± 1.3 (6.0)	5.6 ± 2.4 (5.6)	0.106	5.7 ± 2.5 (5.5)	5.6 ± 2.1 (6.0)	0.952
Current BPN dose (Oct’ 21)	5.8 ± 2.8 (5.2)	6.3 ± 2.3 (6.0)	5.8 ± 2.8 (5.2)	0.269	5.7 ± 2.8 (6.7)	6.2 ± 3.0 (5.1)	0.343
No. of advance doses (Oct’ 21)	9.5 ± 1.1 (10.0)	10.0 ± 0.0 (10.0)	9.5 ± 1.2 (10.0)	0.145	9.5 ± 1.2 (10.0)	9.7 ± 0.8 (10.0)	0.495
Duration on BPN (years)	4.8 ± 2.7 (5.0)	6.8 ± 1.8 (6.5)	4.7 ± 2.7 (5.0)	0.011	4.8 ± 2.7 (5.0)	5.0 ± 2.7 (5.0)	0.559
Distance from OST center (km)	4.9 ± 3.4 (4.0)	3.9 ± 3.0 (3.0)	5.0 ± 2.4 (4.0)	0.109	5.1 ± 3.7 (4.0)	4.3 ± 2.3 (4.0)	0.566
GGZ score	7.6 ± 1.5 (8.0)	3.5 ± 1.2 (3.5)	7.9 ± 0.2 (8.0)	0.000	7.7 ± 1.3 (8.0)	7.5 ± 2.2 (8.0)	0.674

Quality assessment based on the GGZ Thermometer (Table 3) showed high standards being maintained for domains of information (positive rating by 93.7% clients) and results of treatment or guidance (positive rating by 97.2% clients). However, improvement was required for domains of participation (positive rating by 46.5% of clients) and healthcare providers (positive rating by 61.3% of clients). Specifically, in the domain of involvement, most clients (51.4%) were not aware of any treatment plan being drawn up for them or that they were in agreement with the plan. In the healthcare provider domain, 32.4% and 34.5% of clients felt that the providers did not have enough respect for them or showed interest in them, respectively. Group comparison based on GGZ score showed that unsatisfied clients (GGZ score <6) differed from satisfied clients (GGZ score ≥6) in some of the items on the scale, but not all. These were information and results of treatment or guidance domains (p=0.009 and 0.016, respectively). Hence, the overall unsatisfactory rating (GGZ score <6) was not based on the domains of participation and healthcare provider, in which the center lacked.

**Table 3:** Quality assessment as per GGZ Thermometer *Chi-square test used; Fisher’s exact test used where ≥20% expected cell counts were less than 5. GGZ thermometer: Geestelijke Gezondheidszorg (Dutch, Mental Healthcare) Thermometer for Appreciation by Clients [6]

Question no.	Parameters/ Questions	Response (Yes/ No)	Total sample (N=142)	GGZ overall score		p value*	Domain total		p value*
				<6 (n=8)	≥6 (n=134)		<80% (n=34)	≥80% (n=108)	
			n (%)	n (%)	n (%)		n (%)	n (%)	
Domain 1 – Information									
D1	Was the overall information provided optimal?	Yes	133 (93.7)	5 (62.5)	128 (95.5)	0.009	26 (76.5)	107 (99.1)	0.000
		No	9 (6.3)	3 (37.5)	6 (4.5)		8 (23.5)	1 (0.9)	
Q1	Have you received enough information about the treatment and counseling options of the institution?	Yes	136 (95.8)	6 (75)	130 (97)	0.037	29 (85.3)	107 (99.1)	0.003
		No	6 (4.2)	2 (25)	4 (3)		5 (14.7)	1 (0.9)	
Q2	Have you received sufficient information about the approach to your treatment and guidance?	Yes	136 (95.8)	6 (75)	130 (97)	0.037	29 (85.3)	107 (99.1)	0.003
		No	6 (4.2)	2 (25)	4 (3)		5 (14.7)	1 (0.9)	
Q3	Have you received sufficient information about the expected result of your treatment or guidance?	Yes	134 (94.4)	6 (75)	128 (95.5)	0.066	27 (79.4)	107 (99.1)	0.000
		No	8 (5.6)	2 (25)	6 (4.5)		7 (20.6)	1 (0.9)	
Domain 2 – Participation									
D2	Was your overall participation in treatment or guidance optimal?	Yes	66 (46.5)	2 (25)	64 (47.8)	0.285	0	66 (61.1)	0.000
		No	76 (53.5)	6 (75)	70 (52.2)		34 (100)	42 (38.9)	
Q4	Were you able to participate in the decision-making process about the treatment or guidance you would receive?	Yes	120 (84.5)	6 (75)	114 (85.1)	0.610	19 (55.9)	101 (93.5)	0.000
		No	22 (15.5)	2 (25)	20 (14.9)		15 (44.1)	7 (6.5)	
Q5	Has a treatment or counseling plan been drawn up?	Yes	69 (48.6)	2 (25)	67 (50)	0.276	0	69 (63.9)	0.000
		No	73 (51.4)	6 (75)	67 (50)		34 (100)	39 (36.1)	
Q6	Have you agreed to your treatment or counseling plan?	Yes	69 (48.6)	3 (37.5)	66 (49.3)	0.719	2 (5.9)	67 (62.0)	0.000
		No	73 (51.4)	5 (62.5)	68 (50.7)		32 (94.1)	41 (38.0)	
Domain 3 – Caregiver									
D3	Did the caregiver meet optimal standards overall?	Yes	87 (61.3)	4 (50)	83 (61.9)	0.711	1 (2.9)	86 (79.6)	0.000
		No	55 (38.7)	4 (50)	51 (38.1)		33 (97.1)	22 (20.4)	
Q7	Do you consider the care provider to be sufficiently knowledgeable (good at his/her profession)?	Yes	129 (90.8)	6 (75)	123 (91.8)	0.158	24 (70.6)	105 (97.2)	0.000
		No	123 (9.2)	2 (25)	11 (8.2)		10 (29.4)	3 (2.8)	
Q8	Were you able to trust the care provider sufficiently?	Yes	134 (94.4)	7 (87.5)	127 (94.8)	0.379	26 (76.5)	108 (100)	0.000
		No	8 (5.6)	1 (12.5)	7 (5.2)		8 (23.5)	0	
Q9	Did the care provider show (shows) sufficient respect for you?	Yes	96 (67.6)	5 (62.5)	91 (67.9)	0.714	4 (11.8)	92 (85.2)	0.000
		No	46 (32.4)	3 (37.5)	43 (32.1)		30 (88.2)	16 (14.8)	
Q10	Did you find the care provider sufficiently interested in you and your opinion?	Yes	93 (65.5)	5 (62.5)	88 (65.7)	1.000	2 (5.9)	91 (84.3)	0.000
		No	49 (34.5)	3 (37.5)	46 (34.3)		32 (94.1)	17 (15.7)	
Domain 4 – Result of the treatment or guidance									
D4	Was the overall result of treatment or guidance optimal?	Yes	138 (97.2)	6 (75)	132 (98.5)	0.016	30 (88.2)	108 (100)	0.003
		No	4 (2.8)	2 (25)	2 (1.5)		4 (11.8)	0	
Q11	Was the treatment or supervision plan carried out as desired?	Yes	138 (97.2)	6 (75)	132 (98.5)	0.016	30 (88.2)	108 (100)	0.003
		No	4 (2.8)	2 (25)	2 (1.5)		4 (11.8)	0	
Q12	Do you think the treatment or guidance is the right approach for your problems or complaints?	Yes	141 (99.3)	7 (87.5)	134 (100)	0.056	33 (97.1)	108 (100)	0.239
		No	1 (0.7)	1 (12.5)	0		1 (2.9)	0	
Q13	Have you gained more control over your problems or complaints as a result of the treatment or guidance?	Yes	142 (100)	8 (100)	134 (100)	-	34 (100)	108 (100)	-
		No	0	0	0		0	0	
Q14	Have you made sufficient progress as a result of the treatment or supervision?	Yes	141 (99.3)	7 (87.5)	134 (100)	0.056	33 (97.1)	108 (100)	0.239
		No	1 (0.7)	1 (12.5)	0		1 (2.9)	0	
Q15	Are you better able to do things that are important to you because of the treatment or guidance?	Yes	142 (100)	8 (100)	134 (100)	-	34 (100)	108 (100)	-
		No	0	0	0		0	0	
Q16	Can you deal better with people and situations that you had problems with before because of the treatment or guidance?	Yes	142 (100)	8 (100)	134 (100)	-	34 (100)	108 (100)	-
		No	0	0	0		0	0	
Miscellaneous									
Q20	Would you recommend someone else to seek help from this institution?	Yes	130 (91.5)	3 (37.5)	127 (94.8)	0.000	29 (85.3)	101 (93.5)	0.159
		No	12 (8.5)	5 (62.5)	7 (5.2)		5 (14.7)	7 (6.5)	

Correlation analysis (Table 4) shows that after controlling for age, current BPN dose, and GGZ score, duration on BPN was positively correlated (moderate correlation, r=0.433, p=0.000) with the initial or starting BPN dose of clients on OST. Further, controlling for age, Initial BPN dose, and duration on BPN, the GGZ score, i.e., overall quality of OST center, had a weak negative correlation with current BPN dose, signifying that current BPN dosage was a negative indicator of quality.

**Table 4:** Correlations analysis *Correlation significant at the 0.05 level (2-tailed). **Correlation is significant at the 0.01 level (2-tailed). †Controlled for Age, Current BPN dose and GGZ score. ‡Controlled for Age, Initial BPN dose and Duration on BPN. §Controlled for Age, Initial BPN dose and Current BPN dose. BPN: buprenorphine+naloxone; OST: opioid substitution therapy; GGZ: Geestelijke Gezondheidszorg (Dutch, Mental Healthcare) Thermometer for Appreciation by Clients [6]

Zero-order correlations (Kendall’s tau b)														
Categories		Age		Initial BPN		Current BPN			Duration on BPN		Distance from OST center		GGZ score	
Age		1		0.063		-0.031			0.123*		-0.017		-0.039	
Initial BPN		0.063		1		0.068			0.353**		0.074		-0.090	
Current BPN		-0.031		0.068		1			0.050		-0.026		-0.136*	
Duration on BPN		0.123*		0.353**		0.050			1		0.103		-0.155*	
Distance from OST		-0.017		0.074		-0.026			0.103		1		-0.021	
GGZ score		-0.039		-0.090		-0.136*			-0.155*		-0.021		1	
Partial non-parametric correlations														
	Duration on BPN		p value †				GGZ score	p value ‡				GGZ score		p value §
Initial BPN	0.433		0.000**		Current BPN		-0.170	0.046*		Duration on BPN		-0.162		0.057

**Figure 2:**
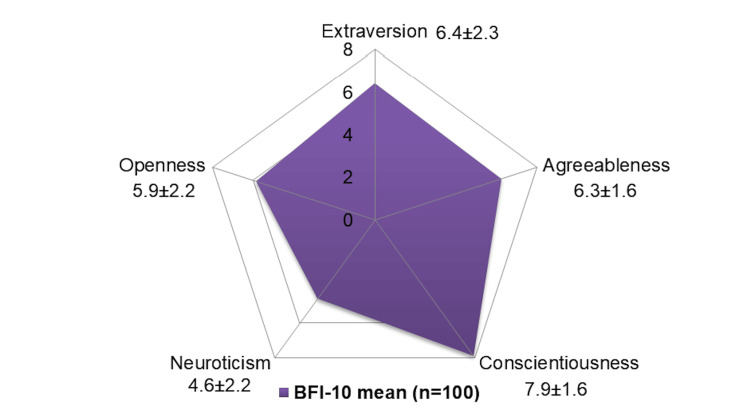
Personality assessment of clients mean±SD mentioned below each category BFI-10: Big Five Inventory- Brief

Since satisfied clients had lesser duration on OST and had a lower, rather than a higher, current BPN dose, personality factors were thought to be at play. For this purpose, the BFI-10 scale was applied after obtaining preliminary results. A total of 100 out of 142 clients were available in the timeframe of post-hoc personality assessment and consented to the same. Personality assessment (Figure 2) showed high conscientiousness and low neuroticism amongst the Big Five traits (Wilcoxon one-sample Signed-rank test used; p≤0.001 for all comparisons). 

## Discussion

Given the single-center, cross-sectional nature of the sample and the exclusion of irregular or terminated clients, these findings represent the experiences of regular attendees rather than the entire OST population.

Socio-demographic variables

The clients had a mean age of 40.4 ± 10.0 years, all male, most were married, had regular partners, were educated till matriculation, were full-time employed, and were referred by TI workers. Only a few clients were positive for any viral markers, had a positive psychiatric history, or had a co-morbid substance use. A previous 2021 survey from Georgia, United States, similarly found that most enrolled clients were 37-50 years of age, male, married, and educated [9]. However, it found that 48% were unemployed, while 85% tested positive for viral markers, in contrast to the current study. Demographic differences could account for these differences. In inter-group comparisons, no difference was found in socio-demographic variables, starting or current BPN dose. 

Mean score variables

Largely, the OST center received a good rating. Overall, the mean duration on BPN was 4.8 ± 2.7 years. Previous literature found that more than half were satisfied with the OST service and its physical characteristics [9]. Mean initial dose was 4.6 mg/day as compared to 5.6 ± 2.4 mg in our study [10]. The average duration of clients’ involvement was six years, which stood somewhat close to that in the satisfied group in our study (6.8 ± 1.8 years). The majority went to the OST site every five days, and a few received daily doses, almost like the current study, with most clients presenting every three days [9]. Unsatisfied clients (GGZ score <6) had a longer duration on OST than satisfied clients; however, given the small size of the <6 group, this pattern should be interpreted cautiously. This observation may reflect heterogeneous client expectations rather than any causal effect of treatment duration. Studies regarding the duration of OST have split views [1,11,12]. Many raise points on the questionable efficacy and dissatisfaction of clients for long-term maintenance, while others quote the risk of relapse after short-term treatment. Further, ‘recovery-oriented practice’, which has been the ultimate aim of OST, supports treatment discontinuation only after the set predetermined goals have been met, rather than a fixed OST duration. As the current study quotes results from one center, a large-scale study may be planned to elicit such points.

Quality assessment based on the GGZ thermometer

Based on the questionnaire, the center was satisfactory in quality standards regarding domains of provision of optimal information’ and ‘overall result of treatment or guidance', while lacking in domains of ‘overall participation’ and ‘caregiver’ (particularly not enough respect or interest shown). The extremely high positive responses in several items, particularly in treatment outcome questions, may reflect some social desirability bias rather than uniformly high quality, which needs consideration. Regarding participation, most were unaware of a treatment plan being drawn up for them. The coexistence of high overall satisfaction scores with low involvement and caregiver ratings likely reflects structural and cultural factors in OST settings, where patients may prioritize medication access and stability over participatory elements of care. However, it also indicates a clear need to strengthen relational and collaborative aspects of OST care. Stigma of being associated with substance use leading to a lower overall expectation from the service, the tag of being drug-free, reduced involvement in the criminal justice system, and personality factors might be some factors contributing to the same. However, these factors need to be explored in future research.

In the Georgian survey, more than half of the clients reported the OST site to be of good quality, information received from staff as sufficient, and their decisions about initiating or terminating enrollment were influenced by the staff’s behavior [9]. This stands in line with the current study and hence highlights the importance of caregivers/staff and participation of clients in the treatment process. More than 90% reported the BPN dose to be sufficient. Unlike ours, more than half of the clients in the survey reported not being safe at the OST site, had problems with video surveillance, and a breach of confidentiality. Even more were unsatisfied with the psychosocial support available on the site (63%), and 38.8% indicated expected availability of a psychologist service.

Safety at the OST site was not a factor in the current study, possibly because the center was inside the hospital premises and staff employed strict measures on suspicion of diversion or other malintent. Break in privacy was thought by the investigators to be a matter of concern for the clients in current study too; however, it was not so, which may be because of the center's location within the general hospital setting, or that since many clients are enrolled with the center using snowball technique by the TI workers, so that may have allayed confidentiality/privacy concerns. Lastly, satisfactory psychosocial support at the center was required by clients in the current study too, but in much less prevalence than the United States survey (4.1% versus 63%, respectively) [9]. It seems not to be the case that psychosocial support at this study’s center was optimal, considering that client participation and healthcare provider standards were lacking. Enhancing awareness of psychosocial stressors as a cause for substance initiation or continuation may raise the proportion of clients requiring satisfactory psychosocial support.

The open-ended comments/suggestions that came from the clients were in the order of: option for advance doses > difficulty in daily visit > long waiting time > add-on medications. For the former two, various reasons exist, including unsuitable job timings, financial problems reaching the center, frequent out-of-town visits, and desirability of control over medication. The desired changes expressed by clients in the Georgian survey matched somewhat with the current study- need for practice of taking medicines at home, geographical accessibility, avoiding travel cost and time, enormous queues, confidentiality breach by standing in queues, increase in working hours of one of the central clinics, and problem with video surveillance of the center [9]. Another study stated that retention in treatment was better with the availability of take-home/advance doses [13]. 

Correlation analysis

The initial BPN dose positively correlated with duration of being on BPN, while the current BPN dosage was a negative indicator of the quality of the OST center. However, the effect size was small and hence may be considered exploratory. A systematic review by O'Connor et al. for methadone, BPN, and mixed cohorts could trace higher retention with higher methadone dose, but mixed reviews for BPN, increased BPN dose, and better retention in one, and negative effects in another cohort [13]. For the mixed OST cohort, better retention was noted among those receiving high and medium doses than lower doses. Another study stated that a higher induction dose and rapid escalation of the BPN dose (average 16 mg at day 28) reflected better satisfaction and retention, even when controlling for baseline characteristics [14]. Contrasting results in the current study could again be directed towards personality factors and stigma of continuing OST. 

Personality assessment

After reviewing the preliminary results on the GGZ thermometer and noticing the negative attitudes toward OST continuation in some individuals, it was discussed internally that personality may play a role in attitudes towards OST. The post-hoc personality assessment, conducted only in those available and consenting, suggested higher conscientiousness, but this remains exploratory and may be subject to selection bias. Previous studies from north India, including Punjab, showed that clients and some psychiatrists had negative views on opioid maintenance with BPN, considering it to be addictive [15,16]. Past research also suggests that higher conscientiousness moderates substance use [17]. Therefore, individuals with high conscientiousness who view BPN as addictive (a preconceived notion) might have valid reasons for wanting to taper OST early. Additionally, high conscientiousness, associated with goal-oriented behavior, may prompt clients to seek shorter OST durations due to perceived self-control. However, this explanation does not account for BPN's pharmacodynamics, warranting further research to explore the psychological effects of BPN maintenance. Summarizing, given the cross-sectional design, exploratory analyses, and selective sample of regular attendees, the findings should be interpreted as descriptive insights rather than evaluative judgments of treatment quality. The results highlight service-level areas for improvement rather than establishing causal mechanisms.

Limitations

Being a single-center, cross-sectional evaluation of regular attendees, with purposive sampling and exclusion of dropouts (although small), irregular attendees, and high-risk (‘blacklisted’) clients, the findings are not generalizable. The Punjabi translation of the GGZ Thermometer was not formally back-translated or culturally validated, and therefore, the scale’s psychometric properties in this population remain preliminary. Group classifications and threshold definitions were exploratory and not based on a priori hypotheses, and the small subgroup sizes (e.g., n=8) limit statistical power. Ceiling effects in several GGZ items raise the possibility of social desirability bias or measurement insensitivity. Correlation analyses reflect weak associations and may not be interpreted causally. The post-hoc personality assessment was exploratory and subject to selection bias. Overall, the results should be viewed as a descriptive service-evaluation snapshot rather than a definitive measure of OST quality. These findings provide preliminary insights but require confirmation through larger, longitudinal studies to establish causal links.

## Conclusions

This study demonstrates that while clients on long-term OST report high satisfaction with information provision and treatment outcomes, important gaps persist in shared decision-making and the interpersonal aspects of care. These findings underscore the need for structured patient-engagement practices, improved provider communication training, and routine psychosocial support alongside pharmacotherapy. The weak associations between dose, duration, and satisfaction are exploratory and should not guide treatment decisions but highlight areas for future longitudinal research. The post-hoc personality observations also remain preliminary but suggest that patient-level psychological factors may warrant systematic assessment in future studies. Overall, this service-evaluation identifies clear opportunities to strengthen patient-centered OST delivery in similar Indian clinical settings, and it provides a foundation for more rigorous, validated, and inclusive research moving forward.
